# Associations of Serum Cathepsin K and Polymorphisms in *CTSK* Gene With Bone Mineral Density and Bone Metabolism Markers in Postmenopausal Chinese Women

**DOI:** 10.3389/fendo.2020.00048

**Published:** 2020-02-13

**Authors:** Li-hong Gao, Shan-shan Li, Hua Yue, Zhen-lin Zhang

**Affiliations:** Shanghai Clinical Research Center of Bone Diseases, Department of Osteoporosis and Bone Diseases, Shanghai Jiao Tong University Affiliated Sixth People's Hospital, Shanghai, China

**Keywords:** cathepsin K, bone mineral density, bone metabolic markers, SNPs, postmenopausal women

## Abstract

Cathepsin K plays an important role in bone resorption. The reports of the association of serum cathepsin K with bone mineral density (BMD) and bone turnover markers are conflicting and the role of serum cathepsin K as a bone turnover marker is unclear. The aims of the study were as follows: (1) to investigate the association of serum cathepsin K with BMD and markers of bone turnover and (2) to evaluate the correlations of single-nucleotide polymorphisms (SNPs) within the *CTSK* gene with serum cathepsin K, BMD, and markers of bone metabolism in postmenopausal Chinese women. A cross-sectional study was conducted with 1752 postmenopausal Chinese women. Four tagging SNPs (rs12085336, rs12746973, rs4379678, and rs10847) of the *CTSK* gene were genotyped. Serum cathepsin K of 768 and markers of bone metabolism of 1752 including serum intact PTH, 25-hydroxyvitamin D [25(OH)D], procollagen type 1 N-terminal propeptide (P1NP), and β-CrossLaps of type I collagen containing cross- linked C-telopeptide (β-CTX) were measured. The BMD of the lumbar spine and proximal femur were measured by dual-energy X-ray absorptiometry (DXA). No significant relationship was detected between serum cathepsin K and age, BMI, BMD or bone metabolic markers (all *P* > 0.05) after adjustment for age and BMI. We failed to identify any significant association between the genotypes or haplotypes of *CTSK* and BMD, bone turnover markers, or serum cathepsin K. Neither serum cathepsin K nor *CTSK* gene polymorphisms was correlated with BMD or bone turnover markers. Genetic polymorphisms of *CTSK* may not be a major contributor to variations in the serum cathepsin K or BMD in postmenopausal Chinese women. The results implied that serum cathepsin K may not be viewed as a substitute for bone turnover markers.

## Introduction

Osteoporosis is a systemic skeletal disorder characterized by decreased bone mineral density (BMD) and compromised bone microarchitecture leading to an increased risk of fracture (1). BMD, reflecting the bone strength, is the gold standard for osteoporosis diagnosis and is one of the most important predictors of osteoporotic fracture (2). Measurement of bone turnover markers may reflect the cross-sectional BMD and predict the fracture, however, the results are inconsistent (3–5). Cathepsin K encoded by cathepsin K (*CTSK*) gene is a lysosomal cysteine proteinase involved in bone resorption and is predominantly expressed in osteoclasts. The main mechanism is that cathepsin K has the unique ability to cleave both helical and telopeptide regions of type 1 collagen, which comprises more than 90% of the organic matrix of bone (6). Saftig et al. (7) showed that impaired osteoclastic bone resorption leads to osteopetrosis in *CTSK* deficient mice. Analysis of the bones of *CTSK* knockout mice revealed that demineralization by osteoclasts is intact, whereas matrix degradation is significantly diminished (8). Mutations in *CTSK* gene are the cause of pycnodysostosis, an autosomal recessive disease characterized by osteosclerosis and short stature (9, 10). Specific inhibition of cathepsin K has therefore been a new drug target for diseases that have elevated bone resorption such as osteoporosis. ONO-5334, a low-molecular-weight synthetic inhibitor of cathepsin K, has been shown to increase areal BMD at the hip and spine in postmenopausal osteoporosis (11–13).

Postmenopausal osteoporosis is characterized by increased bone resorption that exceeds bone formation resulting in a high bone turnover state that may be identified by measurement of biochemical markers (14). Recently, serum cathepsin K was introduced as a potential new bone turnover marker. Holzer et al. (15) reported that serum cathepsin K in individuals with multiple non-traumatic fractures was significantly higher than that in those without fractures, suggesting that cathepsin K could serve as a marker for fracture prediction. Meier et al. (16) found that serum cathepsin K appeared to reflect osteoclastic activity in patients with postmenopausal osteoporosis and Paget's disease of bone. However, the results from the study of Adolf et al. (17) which was performed in premenopausal and postmenopausal women indicated that serum cathepsin K levels were not suitable to differentiate women with osteoporosis from healthy subjects. So far, the results regarding the association of serum cathepsin K and BMD or bone turnover markers vary, and the specific conclusions are needed to clarified in different populations. In addition, no reports on the association of serum cathepsin K and BMD or bone turnover markers in Asian people have obtained for now, moreover, no study has been undertaken to simultaneously evaluate the serum levels of cathepsin K and polymorphisms in the *CTSK* gene. Therefore, the main objectives of this study were as follows: (1) to observe the association of serum cathepsin K with both BMD and bone metabolism markers and (2) to investigate the relationships of single-nucleotide polymorphisms (SNPs) of the *CTSK* gene with serum cathepsin K, BMD, and bone metabolism markers in postmenopausal Chinese women.

## Subjects and Methods

### Study Population

The study was approved by the Ethics Committee of Shanghai Jiao Tong University Affiliated Sixth People's Hospital. Women who had been postmenopausal naturally for more than 1 year and were older than 50 years were eligible for the study. All the participants had a physical examination and routine laboratory measurements. Participants who received treatment for osteoporosis, drugs affect bone or vitamin D metabolism, received vitamin D and/or calcium supplementation within the prior 12 months, or had medical complications known to affect bone metabolism were excluded. A total number of 1799 unrelated, independent ambulatory postmenopausal female volunteers were recruited from outpatient clinics for osteoporosis. Twenty-nine subjects were excluded because they had taken alendronate or estrogen replacement therapy, and another 18 subjects were excluded for abnormal serum calcium or phosphorus levels or abnormal liver or renal function. Finally, a total of 1752 postmenopausal women (aged 50–94.9 years) were retained for this study. At the same time, 768 subjects were selected randomly by an online random number generator “wyrand” (https://github.com/wangyi-fudan/wyhash) from the whole study population for serum cathepsin K analysis. All participants signed an informed consent form before inclusion.

### BMD Measurements

The BMD (g/cm^2^) of the lumbar vertebra 1-4 (L1-L4), left femoral neck, and total hip was measured using dual-energy X-ray absorptiometry (DXA) of Lunar Prodigy (GE Lunar Corp). The Lunar device was calibrated daily. The coefficient of variability (CV) of the DXA measurements of the lumbar vertebra, femoral neck, and total hip were 1.39%, 2.22%, and 0.70%, respectively (18, 19). All DXA measurements were conducted by the same well-trained technologist throughout the study.

### Laboratory Tests

Fasting blood samples were obtained from 8:00 to 10:00 AM, serum samples were stored at −80°C until analysis. The bone turnover markers were measured following both the manufacturer's protocol and specialized laboratory assay quality control procedures. Serum calcium (Ca), phosphorus (P), alkaline phosphatase (ALP) were measured using a Hitachi 7600-020 automatic biochemistry analyzer. The intra- and inter-assay CVs were 1.5% and 2.0% for Ca, 2.1% and 2.3% for P, and 2.5% and 4.5% for ALP, respectively. Intact PTH, 25(OH)D, procollagen type 1 N-terminal propeptide (P1NP) and β-CrossLaps of type 1 collagen containing cross-linked C-telopeptide (β-CTX) were measured using an automated Roche electro-chemiluminescence system (Roche Diagnostic GmbH). The intra- and inter-assay CVs, were 1.4% and 2.9% for PTH, 5.7% and 7.3% for 25(OH)D, 2.9% and 3.8% for P1NP, and 2.5% and 3.5% for β-CTX, respectively (20, 21). Serum cathepsin K of 768 subjects selected randomly from the study population was measured using an enzyme immunoassay for the quantitative determination of cathepsin K in serum (Cathepsin K ELISA, Biomedica Medizinprodukte GmbH and Co KG, catalog number BI-20432). The intra- and inter-assay CVs were 4–6% and 6–8%, respectively, and the detection limit was 1.1 pmol/L. This assay has been validated in previous studies (17, 22).

### SNPs Selection and Genotyping

SNPs of the *CTSK* gene were obtained from the dbSNP (http://www.ncbi.nlm.nih.gov/snp/) and HapMap (http://hapmap.ncbi.nlm.nih.gov/) database. SNPs were selected according to the following criteria: (1) minor allele frequency (MAF) higher than 5% in the Chinese Han population; (2) classified as tagging SNPs and with pairwise linkage disequilibrium (LD) exceeding the threshold of 0.8 (*r*^2^ > 0.8). In all, four tagging SNPs (rs12085336, rs12746973, rs4379678, and rs10847) in *CTSK* gene were selected in the study. To genotype the selected tagging SNPs, genomic DNA was extracted and purified using the QuickGene DNA whole blood kit L by Nucleic Acid Isolation System (QuickGene-610L, FUJI FILM, Japan). SNPs were genotyped using the ABI PRISM SNaPshot multiplex kit (Applied Biosystems), an Mx3000p real-time PCR system (Stratagene), and GeneMapper 4.1 (Applied Biosystems).

### Statistical Analysis

The distribution of all continuous variables was assessed and extreme values were excluded. Descriptive statistics were given as the mean ± SD for normally distributed data and as the median and interquartile range for the non-normally distributed data. Natural logarithmic transformation was conducted for skewed variables to approximate normality for subsequent analyses.

Hardy-Weinberg equilibrium for every SNP was checked using the χ^2^ goodness-of-fit statistic. The level of linkage disequilibrium (LD) among *CTSK* SNPs was assessed using Haploview version 4.2. Haplotypes were constructed from the population genotype data using the test implemented in PLINK. Quality control filtering and allele, genotype, and haplotype association tests between the adjusted parameters and SNPs were conducted using the Wald test implemented in PLINK. Multiple linear regression was used to evaluate the association between BMD, bone turnover markers, and serum cathepsin K and genotypes, and the association between BMD, bone turnover markers, and serum cathepsin K and haplotypes. The analyses were based on additive, recessive and dominant models (using BMD, bone turnover markers, and serum cathepsin K as dependent variables) and adjusted for age and BMI.

A multiple linear stepwise regression analysis was performed to assess the independent contribution of the following variables to serum cathepsin K: age, BMI, markers of bone metabolism and BMD. The False Discovery Rate (FDR) method was applied to control family-wise error rate when performing multiple tests. Statistical analyses were performed using SPSS version 19.0 for Windows (SPSS Inc., Chicago, IL, USA). The threshold for the replication significance was set at *P* < 0.05.

## Results

### General Characteristics of the Study Population

The basic characteristics of the whole study population (*n* = 1,752) and the participants for serum cathepsin K analysis (*n* = 768) are presented in Table 1. The participants measured serum cathepisn K had a similar age, 65.15 ± 8.60 (50–93.1) years, with the whole group [65.38 ± 8.98 (50–94.9) years], and a median serum cathepsin K level of 12.29 (interquartile range 8.80–16.22) pmol/L. There was no significant difference between the two groups in the basic characteristics including age, TSM, BMI, BMD of lumber 1–4, femoral neck and total hip, PTH, 25(OH)D, Ca, P, ALP, P1NP, β-CTX, serum albumin, creatinine, or urea nitrogen. In the overall study population of 1752, the subjects with osteoporosis, osteopenia and normal bone mass were 588, 867, and 297, respectively, and the number of that in the *n* = 768 subgroup were 232, 377, and 159, respectively.

**Table 1:** Clinical characteristics of participants in the study.

**Characteristics**	**Subjects with serum cathepsin K (*n* = 768)**	**Total sample (*n* = 1,752)**
Age (year)	65.52 ± 8.44	65.38 ± 8.98
TSM (year)	14.60 (8.90-22.30)	15.00 (9.00-22.60)
BMI (kg/m^2^)	23.43 ± 3.30	23.47 ± 3.29
Lumbar 1-4 BMD (g/cm^2^)	0.89 ± 0.14	0.88 ± 0.14
Femoral neck BMD (g/cm^2^)	0.73 ± 0.11	0.72 ± 0.11
Total hip BMD (g/cm^2^)	0.77 ± 0.12	0.77 ± 0.12
PTH (pg/mL)	41.04 (32.01–52.61)	40.66 (32.06–51.98)
25(OH)D (ng/mL)	21.76 (17.26–26.94)	18.29 (13.29–23.84)
Ca (mmol/L)	2.32 ± 0.10	2.34 ± 0.12
P (mmol/L)	1.16 ± 0.14	1.16 ± 0.15
ALP (U/L)	72.00 (60.00–86.00)	73.00 (62.00–88.00)
P1NP (ng/mL)	57.90 (43.90–75.41)	56.94 (43.36–74.33)
β-CTX (ng/L)	420.00(296.00–559.5)	410.00 (284.00-558.00)
CTSK (pmol/L)	12.29 (8.80–16.22)	

*Normally distributed data are shown as the mean ± SD, data that are not normally distributed data are shown as the median (interquartile range). TSM, time since menopause; BMI, body mass index; BMD, bone mineral density*.

### Associations of Serum Cathepsin K With Markers of Bone Metabolism and BMD

The serum cathepsin K concentrations were measured in 768 participants selected randomly from the whole study population. In the overall population of 1752, we found that both β-CTX and P1NP have a significant relationship with BMD of L1-L4, left femoral neck, and total hip (all *P* < 0.05). In the *n* = 768 subgroup, significant relationship was detected between β-CTX and P1NP and BMD of left femoral neck, and total hip (all *P* < 0.05). However, we failed to identify any significant association between BMD of L1-L4 and β-CTX or P1NP (all *P* > 0.05). Multiple linear regression analysis was performed using serum cathepsin K as the dependent variable and age, BMI, serum β-CTX, P1NP, PTH, 25(OH)D, ALP, BMD of the L1-L4, femoral neck and total hip as the independent variable in the subjects (Table 2). It is found that serum cathepsin K level was positively associated with the serum 25(OH)D of the studied postmenopausal women (*P* = 0.049), however, the significance disappeared after adjustment for age and BMI (*P* = 0.053). No association was found between serum cathepsin K and age, BMI, or between serum cathepsin K and serum P1NP, β-CTX, PTH, ALP, or 25(OH)D, or between the serum cathepsin K and BMD of L1-4, femoral neck or total hip (all *P* > 0.05) (Table 2).

**Table 2:** Correlations between serum CTSK and other anthropometric and clinical data.

	**Standardized β-Coefficients**	***P*-value**	**Standardized β-Coefficients^a^**	***P*-value^**a**^**
Age (year)	−0.034	0.343		
BMI (kg/m^2^)	−0.015	0.673		
Lumbar 1-4 BMD (g/cm^2^)	0.047	0.201	0.045	0.227
Femoral neck BMD (g/cm^2^)	−0.023	0.530	−0.050	0.237
Total hip BMD (g/cm^2^)	−0.009	0.796	−0.027	0.528
β-CTX (ng/L)	0.029	0.432	0.026	0.467
P1NP (ng/mL)	0.041	0.253	0.038	0.293
PTH (pg/mL)	0.017	0.629	0.024	0.519
ALP (U/L)	0.014	0.772	0.016	0.741
25(OH)D (ng/mL)	0.071	**0.049**	0.070	0.053

*Significant values (P < 0.05) are presented in bold*.

a *Adjusted for age and BMI*.

### Allele Frequencies and Haplotype Structures

Four SNPs in the *CTSK* gene of 1752 unrelated postmenopausal Chinese women were genotyped in the study. Information of four SNPs is shown in Table 3. The genotyping success rate for all SNPs exceeded 95%. Overall, 4 polymorphisms of *CTSK* gene had a MAF at least 0.116 in this study and the genotype frequencies of all 4 SNPs were compatible with the Hardy-Weinberg equilibrium (HWE) (*P* > 0.05). LD was measured by Haploview software to calculate the D' value which represent the strength of each pairwise comparison of biallelic SNPs. In *CTSK*, we distinguished one block with high LD (all D'=1.0) containing rs12085336, rs12746973, rs4379678, and rs10847. On the basis of these polymorphisms, significant LD was observed across the gene and we inferred the existence of 5 haplotypes in the block, TGTC, AGCC, AGTT, AATC, and AGTC with frequencies of 0.338, 0.231, 0.204, 0.117, and 0.110, respectively.

**Table 3:** Information of the four studied SNPs.

**SNPs in dbSNP**	**Chr**	**Position**	**Region**	**SNP**	**MAF (this study)**	**MAF (hapmap-HCB)**	**MAF (hapmap-CEU)**
rs12085336	1	150775780	intron 5	A/T	0.338	0.357	0.373
rs12746973	1	150779401	intron 1	G/A	0.116	0.106	0.053
rs4379678	1	150780582	intron 1	C/T	0.233	0.241	0.083
rs10847	1	150782797	5′-flanking	C/T	0.205	0.211	0.225

### Associations Between Genotypes and Haplotypes of *CTSK* and Serum Cathepsin K, Markers of Bone Metabolism and BMD

We investigated the association between four SNPs and five haplotypes of *CTSK* gene and serum cathepsin K in 768 subjects, and the association between four SNPs and five haplotypes and bone metabolism markers and BMD of L1-L4, femoral neck and total hip in 1752 subjects using PLINK. We performed multiple liner regression and adjusted all the variables for age and BMI. Haplotype AGTC of *CTSK* was found to be significantly associated with BMD of L1-4 (*P* = 0.019) (Table 4) and SNP rs12746973 was confirmed to be significantly associated with variations in the serum P1NP levels (*P* = 0.032) adjusted for age and BMI (Table 5). However, both the significant correlation disappeared after applying a FDR correction. No association was found between the other bone metabolism markers or BMD of femoral neck or total hip and any of four SNPs or five haplotypes of the *CTSK* gene (data not shown). In addition, there was no association between any of the four SNPs or five haplotypes of *CTSK* gene and serum cathepsin K levels of the 768 postmenopausal women in our study (Table 6).

**Table 4:** Associations between four SNPs, five haplotypes of CTSK, and the BMD of lumber spine 1-4 in this study.

**SNP**	***BETA***	***R^**2**^***	***P*-value^a^**	**haplotype**	***BETA***	***R^**2**^***	***P*-value^a^**
rs12085336	−0.007	9.775e-005	0.192	AGTT	−0.002	0.0003	0.749
rs12746973	−0.021	5.809e-006	0.193	AGCC	0.001	4.039e-005	0.807
rs4379678	0.009	5.135e-005	0.244	AATC	−0.001	5.899e-005	0.944
rs10847	0.009	0.0062	0.338	TGTC	−0.007	0.0001	0.152
				AGTC	0.018	0.0004	**0.019**

*Significant values (P < 0.05) are presented in bold. BETA regression coefficient, R^2^ r-suqared regression coefficient*,

a *adjusted for age and BMI*.

**Table 5:** Associations between four SNPs, five haplotypes of CTSK, and serum P1NP in this study.

**SNP**	***BETA***	***R*^**2**^**	***P*-value^a^**	**haplotype**	***BETA***	***R*^**2**^**	***P*-value^a^**
rs12085336	0.020	8.368e-006	0.583	AGTT	0.072	0.0015	0.067
rs12746973	−0.219	2.346e-005	**0.032**	AGCC	−0.040	0.0004	0.265
rs4379678	−0.042	0.0004	0.385	AATC	−0.034	2.035e-005	0.483
rs10847	0.082	0.0015	0.187	TGTC	0.011	5.087e-006	0.737
				AGTC	−0.028	0.0002	0.571

*Significant values (P < 0.05) are presented in bold. BETA regression coefficient, R^2^ r-suqared regression coefficient*,

a *adjusted for age and BMI*.

**Table 6:** Associations between four SNPs, five haplotypes of CTSK, and serum CTSK in this study.

**SNP**	***BETA***	***R^**2**^***	***P*-value^a^**	**haplotype**	***BETA***	***R^**2**^***	***P*-valuevalue^a^**
rs12085336	−0.223	0.0011	0.760	AGTT	−0.275	0.0005	0.495
rs12746973	−0.532	0.0004	0.165	AGCC	0.563	0.0014	0.308
rs4379678	1.757	0.0014	0.242	AATC	−0.162	0.0004	0.646
rs10847	−0.900	0.0005	0.094	TGTC	0.384	0.0011	0.383
				AGTC	−0.260	0.0003	0.642

*BETA regression coefficient, R^2^ r-suqared regression coefficient*,

a *adjusted for age and BMI*.

## Discussion

To our knowledge, this is the first study reported a population-based association analysis of both *CTSK* gene polymorphism and serum cathepsin K and BMD, bone metabolic markers. Although *CTSK* has been shown to plays a crucial role in the regulation of bone density by both its link with the monogenic disorder pychodysostosis and by manipulation of *CTSK* expression *in vivo* in animal models, as well as to serve as a predictor of bone loss or non-traumatic fracture, we failed to find significant association between serum cathepsin K and BMD or bone metabolic markers levels. Besides, we were unable to show a clear association between *CTSK* polymorphisms and BMD, serum cathepsin K levels or bone turnover markers in postmenopausal Chinese women.

Because cathepsin K plays a critical role in osteoclast-mediated bone degradation by degrading type 1 collagen and osteonectin, major components of the organic matrix of bone, a correlation between higher serum cathepsin K and osteoporosis, increased bone turnover or osteoporotic fracture might be expected. Results from the previous studies demonstrated that the cathepsin K concentrations in subjects with multiple non-traumatic fractures were significantly higher than that in those without fractures and the cathepsin K levels of patients with osteoporosis and Paget's disease of bone were significantly higher than that of healthy controls (15, 16). Munoz-Torres et al. (23) observed that serum cathepsin K levels were higher in 46 postmenopausal women with osteoporosis (9.4 ± 11 pmol/L) compared with 20 healthy postmenopausal women (6.8 ± 8.1 pmol/L; *P* < 0.01) and 20 pre-menopausal women (6.3 ± 5.0 pmol/L, *P* < 0.01). Besides, baseline concentrations of cathepsin K were negatively correlated with BMD at the femoral neck in osteoporotic women and serum cathepsin K decreases gradually after alendronate treatment. These results suggested that the serum level of cathepsin K could serve as a marker of osteoclast activity and predictor for fracture and BMD. However, in our study, we failed to find any association between serum cathepsin K and BMD of lumbar spine or hip of the participants and that was consistent with the results from Prezelj et al. (24), although they reported that serum cathepsin K levels was significant correlation with change in femoral neck BMD in a population of healthy non-osteoporotic women followed up for 3 years, serum levels of cathepsin K did not correlate significantly with either spinal BMD change or cross-sectional BMD of the spine or femoral neck. The varied results of these studies can be attributed to many explanations. The population sizes were relatively small in these studies, dozens or no more than 200 subjects were included, and the small sample size would limit the power to prove relationships and differences. The participants in these studies were various, including participants with and without osteoporotic fracture, postmenopausal osteoporosis, healthy non-osteoporotic pre-, peri, early postmenopausal women. Therefore, the conditions of bone metabolism of these subjects were various as there would be significantly accelerated bone turnover rate and reduced BMD during the menopausal periods due to the serum estrogen levels dramatically decrease within a short time in women (25). In addition, although cathepsin K is expressed at very high levels in osteoclasts, it is also synthesized in other tissues and may play important roles in processes other than bone resorption. Cathepsin K expression has also been found in normal bronchial epithelial cells (26), thyroid epithelial cells (27), synovial fibroblasts of patients with rheumatoid arthritis (28), breast carcinoma cells (29), osteoblast (30) and chordoma (31). Zhao et al. (32) found that circulating cathepsin K increased in patients with chronic heart failure. Therefore, serum cathepsin K level may not necessarily reflect cathepsin K expressed in osteoclasts and could be affected by other pathophysiologic processes. Besides, Dodds et al. (33) found that cathepsin K processing and polarization occurs as the osteoclast approaches bone, and its activation occurs intracellularly, before secretion into the resorption lacunae and the onset of bone resorption, implying that local factors in the microenvironment may regulate these events, for instance, phosphatidylinositol 3-kinases (34). It is found that the sections of osteoclastoma, which included abundant osteoclasts distant from bone formation sites, showed intense expression of cathepsin K mRNA, high levels of procathepsin K, but negligible levels of mature cathepsin K, and this population of osteoclasts showed no cathepsin activity. It means that serum cathepsin K levels may not reflect the osteoclast numbers and it remains to be determined whether this variable expression pattern reflects transcriptional regulation of the cathepsin K gene potentially at different stages of osteoclast function (33). In addition, BMD assessed by DXA measures the total bone mineral content in a projected area (integral areal BMD) and cannot directly measure other skeletal features that may also contribute to bone strength, such as the relative amounts of cortical and trabecular bone. Further studies are needed to clarify the association between the serum cathepsin K and BMD that measured by quantitative computed tomography (QCT) which could provide a direct measurement of cortical and trabecular volumetric BMD, both of which may contribute to bone strength and fracture risk (35).

Biochemical markers of resorption and formation that provide sensitive and dynamic indices of bone metabolic may be a useful investigation tool for quantifying and monitoring the effects of anti-osteoporotic drugs on bone cells (5). In this study, there was no association detected between serum cathepsin K levels and bone metabolic markers, such as β-CTX or P1NP in postmenopausal Chinese women. This is in line with the results of the studies reported before (15, 17, 22, 24). The results imply that serum cathepsin K may not be viewed as a substitute for current bone turnover markers.

In the study, we failed to find any correlation of serum cathepsin K with age of the postmenopausal Chinese women. This is consistent with the previous published studies (16, 23, 24). It is indicated that aging might not be an influencing factor in serum cathepsin K concentration in postmenopausal women.

Osteoporosis is a complex, common disease that results from genetic and environmental causes. Depending on recent studies, many novel genetic loci have been identified as being associated with BMD or osteoporosis (36–38). Given the increasing body of evidence linking *CTSK* with the regulation of BMD, we hypothesized that variation in *CTSK* can result in consequent variation in BMD. Therefore, we tested *CTSK* polymorphisms for possible association with BMD, bone turnover markers and serum cathepsin K in postmenopausal Chinese women. However, there was no significant association detected between four SNPs or five haplotypes and adjusted BMD of L1-4, femoral neck or total hip in this study. These results were consistent with that of Giraudeau et al. (39), who studied the association of *CTSK* gene polymorphisms with BMD in a large cohort of perimenopausal women (~3000) from Scotland, and were unable to show a clear association between *CTSK* polymorphisms and BMD. It is considered that *CTSK* polymorphisms that induce even minor coding or expression changes may have enough deleterious impact on fitness to induce negative selection of such polymorphisms and prevent them from reaching an appreciable frequency and possibly eliminate them from the population over time (39). This may be the reason why we did not find any coding change SNPs with frequencies above 5%, although we extensively screened the entire *CTSK* gene range and regulatory region. The SNPs in our study were located in intron and 5′-flanking in the *CTSK* gene. However, these SNPs may have strong LD with others within the same exons. Studies have shown that intronic variants may also have functional significance, for example, they could affect the splicing of cryptic exons (40). Another possible reason for failure to observe association of *CTSK* polymorphism with BMD is that unknown environmental factors may be confounding correlation between *CTSK* genotypes or haplotypes and BMD, thereby masking the presence of a significant genetic association. Thus, *CTSK* gene common polymorphism may not be a major contributor to BMD variation in postmenopausal Chinese women, although further studies are needed to prove the findings.

There were some limitations in our study. First, the number of subjects in our study was moderate, especially those who measured serum cathepsin K levels. Due to the high prevalence of osteoporosis in the postmenopausal Chinese women visiting our outpatient clinics, we failed to obtain the results of the differences of serum cathepsin K levels between subjects with postmenopausal osteoporosis and healthy postmenopausal controls. Second, the population studied was limited to postmenopausal women, the results of correlation of serum cathepsin K levels or SNPs of *CTSK* gene with BMD or bone metabolic markers in men and younger women could not be afforded. It is of great importance to investigate the association between serum cathepsin K levels or SNPs of *CTSK* gene with BMD or bone metabolic markers in young healthy population, particularly those who with peak BMD. Apart from that, high prevalence of vitamin D insufficient/deficient in the study subjects, which may have an influence on the findings. Besides, this is a cross-sectional study and could not evaluate the predictor effect of serum cathepsin K on BMD change or the risk of osteoporotic fracture. Additionally, we cannot assess the impact that rare variants in *CTSK* gene might have on BMD in our study because we restricted our analysis to tagging SNPs with an MAF >5%. Future studies are needed to consider other types of polymorphisms such as insertion-deletions, copy number variants, and less common SNPs (<5% MAF) as well as interactions between genes and environmental factors in order to better account for the phenotypic variation in BMD.

In conclusion, the results of our study indicated that serum cathepsin K was not correlated to BMD or bone metabolic markers. In addition, common genetic polymorphisms in *CTSK* gene may not be a major contributor to serum cathepsin K or BMD in postmenopausal Chinese women.

## Data Availability Statement

The datasets analyzed for this study can be found in the European Variation Archive (https://www.ebi.ac.uk/ena/data/view/PRJEB36082).

## Ethical Statement

The study has been approved by the Ethics Committee of Shanghai Jiao Tong University Affiliated Sixth People's Hospital. All procedures performed in the study were in accordance with the ethical standards of the Ethics Committee of Shanghai Jiao Tong University Affiliated Sixth People's Hospital and with the 1964 Helsinki declaration and its later amendments or comparable ethical standards. Informed consent was obtained from all individual participants included in the study.

## Author Contributions

LG and SL conducted the study and analyzed the data. LG wrote the draft manuscript. ZZ and HY supervised the study and revised the manuscript. All authors read and approved the final manuscript.

### Conflict of Interest

The authors declare that the research was conducted in the absence of any commercial or financial relationships that could be construed as a potential conflict of interest.
